# Superconductivity in Bismuth. A New Look at an Old Problem

**DOI:** 10.1371/journal.pone.0147645

**Published:** 2016-01-27

**Authors:** Zaahel Mata-Pinzón, Ariel A. Valladares, Renela M. Valladares, Alexander Valladares

**Affiliations:** 1 Departamento de Materia Condensada y Criogenia, Instituto de Investigaciones en Materiales, Universidad Nacional Autónoma de México, México, D.F., México; 2 Departamento de Física, Facultad de Ciencias, Universidad Nacional Autónoma de México, México, D.F., México; Boston College, UNITED STATES

## Abstract

To investigate the relationship between atomic topology, vibrational and electronic properties and superconductivity of bismuth, a 216-atom amorphous structure (*a-Bi*216) was computer-generated using our *undermelt-quench* approach. Its pair distribution function compares well with experiment. The calculated electronic and vibrational densities of states (eDOS and vDOS, respectively) show that the amorphous eDOS is about 4 times the crystalline at the Fermi energy, whereas for the vDOS the energy range of the amorphous is roughly the same as the crystalline but the shapes are quite different. A simple BCS estimate of the possible crystalline superconducting transition temperature gives an upper limit of 1.3 mK. The e-ph coupling is more preponderant in *a-Bi* than in crystalline bismuth (*x-Bi*) as indicated by the *λ* obtained via McMillan’s formula, *λ*^*c*^ = 0.24 and experiment *λ*^*a*^ = 2.46. Therefore with respect to *x-Bi*, superconductivity in *a-Bi* is enhanced by the higher values of *λ* and of eDOS at the Fermi energy.

## Introduction

The field of superconductivity in Materials Science has evolved enormously, from conventional to unconventional passing through the ill-defined undetermined category [1]. Superconductivity of amorphous bismuth (*a-Bi*), considered conventional, was discovered several decades ago with a critical transition temperature of *T*_*c*_ ∼ 6 K, whereas its crystalline counterpart (*x-Bi*) has not been found yet to superconduct, at least above 10^−2^ K [2]. This put the subject in the foreground decades ago, but then it faded into the background as it was not possible to understand the dichotomy observed in the two phases. For this reason bismuth is an interesting material for studying the influence of the structure-property relations on superconductivity. In the Bardeen, Cooper & Schrieffer (BCS) approach superconductivity is a collective phenomenon that invokes an electron-electron attractive interaction mediated by phonons and the coherent motion of these electronic Cooper pairs. So studying the electronic and vibrational properties of these two bismuth phases may contribute to disentangle the mystery of their superconductivity and may shed light on other amorphous superconducting systems where the crystalline counterparts have not yet been found to be superconductors. Like all theories, the BCS approach has advocates and opponents, and the discovery of the so called high *T*_*c*_ superconductors has cast doubts on the mediating mechanism necessary to generate the electron pairs, since it is argued that according to the BCS theory the superconducting transition temperature would be, at best, of the order of the Debye temperature of the material, neglecting the important contribution of the electron density of states at the Fermi level. Nevertheless it is not uncommon to accept the BCS formalism, based on the phonon mediated electron-electron attraction for “normal” low *T*_*c*_ superconductors, since it has proven to be a predictive and accurate theory in this realm. Despite its successes the BCS theory has not been able to foretell which materials would be superconductors and what their superconducting transition temperatures would be due to the difficulty in calculating the e-ph coupling potential from first principles. In this work we report upper bound values for the transition temperature of a given phase if this temperature is known for the other phase and if we can calculate the electronic and vibrational properties of both phases.

The structure of amorphous materials has also been studied for many years but, because of their disordered atomic topology, the relevance of their structure on the electronic and vibrational properties is difficult to explore. Vibrational properties of some amorphous materials have been studied experimentally but in general these measurements are difficult to perform and the same applies to the electronic properties. Also, the manner in which these amorphous samples are generated varies widely: some are produced by chemical vapour deposition; others are generated by ionic bombardment of otherwise crystalline samples until arguably amorphicity appears, or by sudden freezing from the melt. Simulationally, most attempts consider liquefying the samples and then cooling them below their melting points which in general leads to samples with some remnants characteristic of the liquid state. For example, computer generation of amorphous silicon by this approach leads to the presence of atomic overcoordination since in the liquid state, silicon is metallic and overcoordinated with respect to the solid, amorphous or crystalline [3]. This is also the case for germanium and in general, if the liquid phase has different properties from the solid phase, some remnants from the liquid state is observed in the final computationally-generated amorphous structure; floating bonds are a case at hand [4]. To avoid this we devised a new approach that we call the *undermelt-quench* process that consists of heating “crystalline” unstable supercells with the correct density, to temperatures just below the melting value of the real material. A cooling ramp is then applied to the disordered sample with a cooling rate that has the same absolute value as that of the heating ramp and, if necessary, relaxing cycles are applied so that at the end, the energy optimization process leads to an amophous structure that is in a metastable state. A structural property frequently determined for amorphous materials is the radial or pair distribution function (PDF or *g*(*r*)), obtained experimentally by diffraction (neutron, electron or X- ray) methods. A drawback is that since they are averaged one-dimensional quantities a procedure is involved that leads to results non-univocally related to the topology; in other words, it is argued that some disordered topologies lead to indistinguishable PDFs. Clearly there is a need to computer-generate reliable amorphous structures that have PDFs in agreement with experiment and that allow the calculation of important physical properties to compare with experiment and to make predictions. Here we present results that indicate that we have done this for the case of bismuth since we have computer generated an amorphous sample, have calculated PDFs that agree with experiment, and have predicted the electronic and vibrational properties of both phases to see the influence on their superconducting properties.

Since 1950 several authors [5–8], have experimentally studied disordered samples of bismuth and have determined the corresponding PDFs. In general, most authors agree that the structure of *a-Bi* is very similar to the corresponding liquid, but it was not until 1969 when Fujime [2] characterized and gave the following arguments to say that his bismuth sample was amorphous: the PDF obtained by Fujime had 3 peaks, the first one located at 3.28 Å, the second one at 4.5 Å and the third at 6.5 Å; it has a coordination number of *N* = 5.6, where *N* was obtained by integrating a Gaussian-like curve adjusted to the first peak of *J* (*r*) = 4*πr*^2^*g* (*r*). Since we computer-generate an amorphous structure, realistic quantum mechanical “forces” have to be calculated using proven quantum-mechanical methods like the ab initio Density Functional Theory (DFT) approach. A shortcoming is that since the method is so computer-intensive, the size of the structures (periodic amorphous supercells) that can be reasonably used for “finite” time calculations is restricted to a few hundred atoms and thereby only self-averaging [9] properties can be reliably calculated. This is the case for PDFs, densities of states, both electronic and vibrational, optical properties and the like. In previous papers, optical gaps have been calculated using a Tauc-like approach that leads to results in agreement with experiment [10].

## Methods

### Structural simulation

Computationally, we perform simulated annealing processes using the FASTSTRUCTURE code (More detailed description in the Specific Annotations section) and the Harris Functional, a Density Functional Theory (DFT) calculation. Also the Local Density Approximation (LDA), with the parameterization of Vosko, Wilk and Nusair (VWN) [11] is used throughout, although several arguments and counterarguments have been published in the literature concerning their use for certain materials. The calculations are carried out with an all electron basis, within the frozen core approximation. Since bismuth has 83 electrons the valence states are described *via* a minimal basis set of “finite-range” atomic orbitals with a cutoff radius of 5 Å, which is large enough to include fourth neighbours in the crystalline structure. This value for the cutoff was chosen as a compromise between cost and accuracy; as was taking the integration mesh as coarse. The forces are calculated using rigorous formal derivatives of the expression for the energy in the Harris functional [12], as discussed by Lin and Harris [13]. The amorphous structures are computer-generated using a slight modification of the *undermelt-quench* method, starting from an unstable crystalline diamond-like supercell with 216 atoms of bismuth (*a-Bi*216) and a volume of 7645.37 Å^3^ (19.7 Å per edge) [14]. The parameters of the starting structure are adjusted to assure that the interatomic distances are close to those in *x-Bi* and that the density is close to 9.8 g/cm^3^, the density of *x-Bi*, so the final supercell is in the solid state. Since *x-Bi* has a rhombohedral structure, the diamond-like structure is unstable and will disorder readily as the temperature is raised. At temperatures just below the melting point the structure is totally disordered but upon cooling the tendency to crystallization appears so the time step has to be chosen adequately to avoid this process. The simulated annealing processes are carried out as follows: the initial diamond-like supercell is heated form 300 K to 540 K (4.5 K below the bismuth’s melting point) in 100 computational steps. Then it is quenched from 540 K to (close to) 0 K in 225 steps, in order to maintain the same temperature (absolute) rate for the heating as for the cooling. In the cooling process, as the temperature is lowered the disordered structure gets into a metastable topology that leads to an amorphous state. The total simulated annealing procedure lasts about 10 ps. Once the processes are finished, a geometry optimization using DMol^3^ is performed to guarantee that the amorphous structure is at an energy minimum, representing a metastable amorphous solid. When using DMol^3^, we use the computation time scales with a high power of the cutoff radius, because the time-limiting factor of the runs is the number of three-center integrals that have to be computed using the weight-function method of Delley [15, 16] with correction for the dependence of the mesh on the nuclear coordinates. In all cases periodic boundary conditions are utilized. For the *x-Bi* we take the structure given by Wyckoff [17] and repeat it 6 × 6 × 3 times to obtain a 216-atom crystalline supercell (*x-Bi*216). This was done to have the same number of atoms per supercell so the properties calculated for *a-Bi* and *x-Bi* can be directly compared. We then proceed to calculate, first, the radial distribution function *g*(*r*) and compare it to experiment. If simulation and experiment agree then we proceed to calculate the electronic and vibrational densities of states, eDOS and vDOS respectively, of both the crystalline sample and the amorphous one.

### Electronic and vibrational simulations

In the past there was a proposal to approach the difficult problem of calculating the electronic structure of disordered materials, [18]. This method is based on a parameterization of local topologies that are then ensemble- averaged. To calculate the density of states, they introduce the muffin-tin approximation and use multiple scattering theory. In our approach we first generate the atomic topology of the material using our *undermelt-quench* approach and then calculate the properties using density functional first principles techniques. Since we use a supercell, electron energy levels are obtained and the density of electronic states calculated by counting the number of states per unit energy. In this work vibrational frequencies are calculated with *DMol*^3^ for the 216-atom crystalline and amorphous supercells; the method used was the finite displacement approach. The curves were smoothed and normalized so that the number of frequencies equals 3*N*_*A*_ where *N*_*A*_ is the number of atoms in the supercells. In order to compare our crystalline simulational results with experiment, we built the vDOS for *x-Bi* digitizing the points on the dispersion curves collected from several experiments [19] and processed it in the same manner as our data. We also compare our data with the experimental vDOS reported for a temperature of 77 K [20].

To obtain the electronic and vibrational properties a single-point energy calculation has to be carried out first. These single-point energy calculations are performed with the DMol^3^ code in the Materials Studio suite using a double-numeric basis set and a finer mesh within the VWN approximation. Also, an unrestricted spin-polarized calculation for the energy was carried out, leaving out the Harris functional approach. Since bismuth is a heavy element with many electrons, the density-functional semi-core pseudo potential (DSPP) approximation was used [21]. This pseudo potential has been investigated by Delley [21] where an all electron calculation is compared to the DSPP; the rms errors are essentially the same for both methods, 7.7 vs 7.5. Scalar relativistic corrections are incorporated in these pseudopotentials, essential for heavy atoms like Bi. Since DSPPs have been designed to generate accurate DMol3 calculations, it is expected that their use represents a good approximation; evidently, considerations of symmetry were left out for the amorphous, but also for the crystalline. The resulting crystalline eDOS and vDOS were compared with experimental data when available, to benchmark our approach; this would validate our results for the amorphous supercell. We then compare the results for the amorphous and the crystalline cells and through this comparison suggest a reason as to why the amorphous and crystalline phases have different superconducting properties. We also infer a possible upper bound value for the hitherto unknown superconducting transition temperature of *x-Bi*. (A more detailed description can be found in the Specific Annotations section).

## Results and Discussion

### Structure

**Fig 1:**
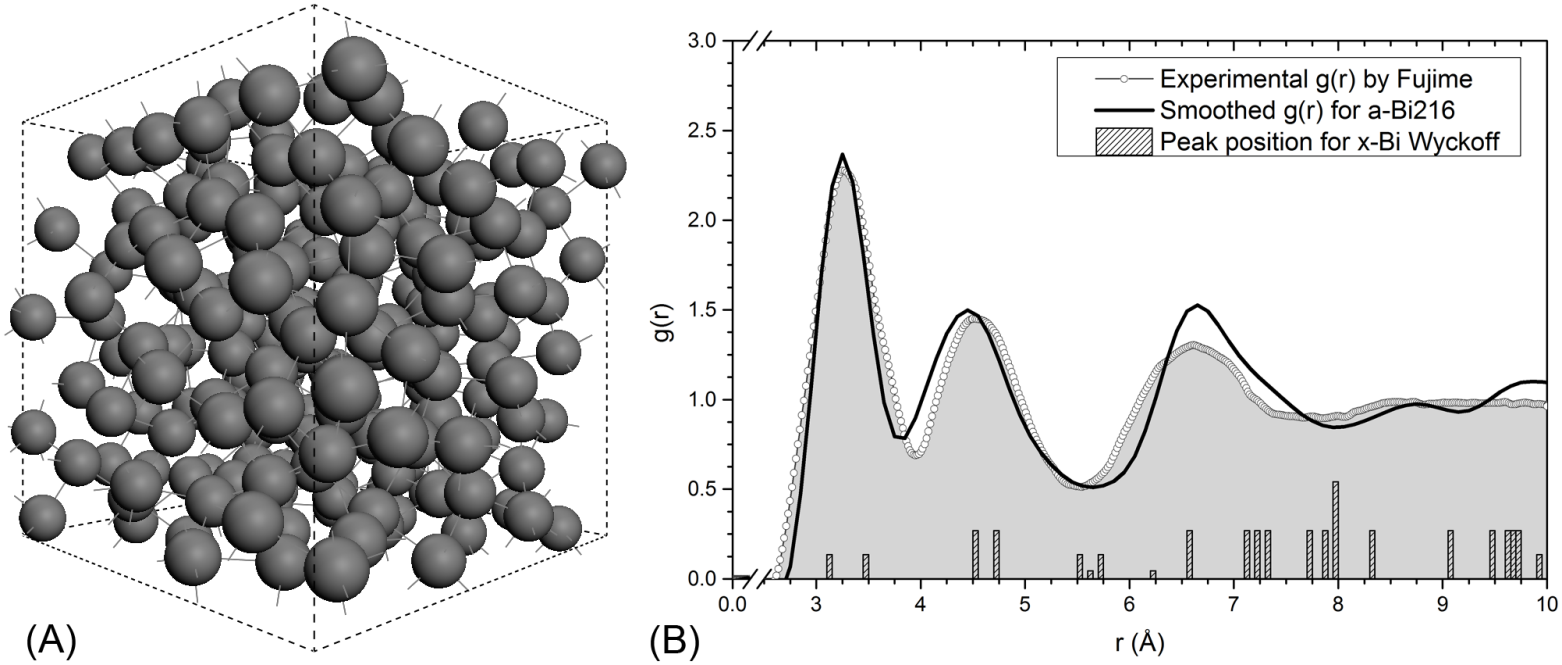
Structural analysis of simulated *a-Bi*. (A) Computer-generated amorphous supercell of bismuth, *a-Bi*216. (B) Computational *g*(*r*) for *a-Bi*216 compared with the experimental results obtained by Fujime [2]. Positions of the peaks for the rhombohedral *x-Bi* are included for contrast with the broader, amorphous ones.

As mentioned above, we obtain a 216-atom amorphous structure of bismuth (*a-Bi*216) by simulated annealing a 216-atom unstable “crystalline” supercell, using a slight modification of the *undermelt-quench* approach proposed by Álvarez *et al*. [3]. To validate the simulations the PDF of the resulting amorphous structure is directly compared with the experimental curve; the computational results for the *a-Bi* structure are shown in Fig 1A which reveals an exceptional agreement with the experimental PDF. Positions and relative errors for the first three peaks of the *g*(*r*) obtained in this work and compared to Fujime’s PDF, are as follows: The relative error for the first peak position (3.25 Å) is 0.91%, for the second peak position (4.45 Å) is 1.11%, and for the third (6.65 Å) 2.26%. This indicates that the amorphous structures generated possess PDFs in agreement with experiment and that calculating electronic and vibrational properties for these structures would be a real test to validate the aforementioned agreement. Something to be noted is that the position of the first amorphous peak (3.25 Å) is at almost the average distance of the first two crystalline peaks (3.11 Å and 3.45 Å). This implies that the first two crystalline peaks coalesce into a single amorphous peak. A similar situation occurs for the second amorphous peak where the third and fourth crystalline peaks coalesce to give rise to the second broaden amorphous neighbors.

Shoulders in the first and second peaks of *g*(*r*) are commonly encountered in metallic glasses, but *a-Bi* does not have marked shoulders due perhaps to its peculiar bonding, somewhat covalent and somewhat metallic, as Ley *et al*. [22] point out. These shoulders in metallic glasses seem to be due to the metallic bond and the characteristic icosahedral geometries that appear in the amorphous structures [23], so the absence of these geometric structures may be attributed to the absence of a completely metallic bond giving washed-out shoulders for the *g*(*r*) of *a-Bi* and a lower coordination number than for amorphous metallic systems. Also, Fujime’s assertion that the *g*(*r*) for the amorphous is similar to that of the liquid may be due to the lack of formation of icosahedral structures in the solid phase if full metallic bonding is absent.

**Fig 2:**
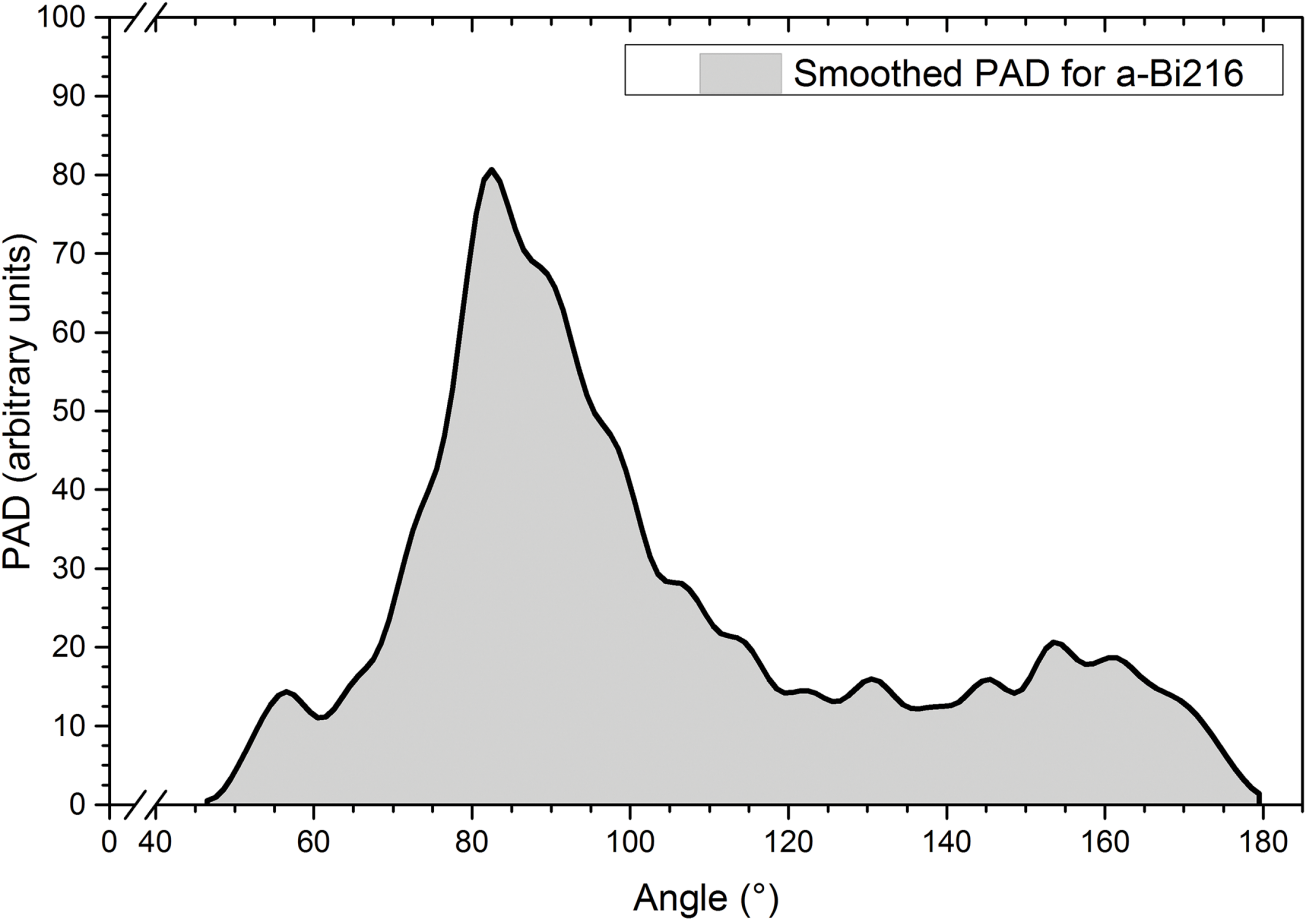
PAD for the *a-Bi*216 supercell. The nearest neighbor values of the plane angle in *x-Bi* are 89° and 91°, whereas the maximum for the amorphous cell occurs at 82.5°. Notice the shoulder located at 56.5° which is reminiscent of angles in an equilateral triangular structure. The bump at about 155° may be due to non-planar surfaces bounding deformed cubes in this amorphous structure.

The Plane Angle Distribution (PAD) for the *a-Bi*216 supercell shows that the maximum is located at 82.5°, a value relatively close to those of the first neighbors in *x-Bi*, 89° and 91°, see Fig 2. The shape of the PAD calculated here is very different from the typical PAD obtained for amorphous metals since for these metallic materials, the distribution displays two preponderant peaks located at about 60° and 116° [24]. (More detailed description in the Specific Annotations section)

**Fig 3:**
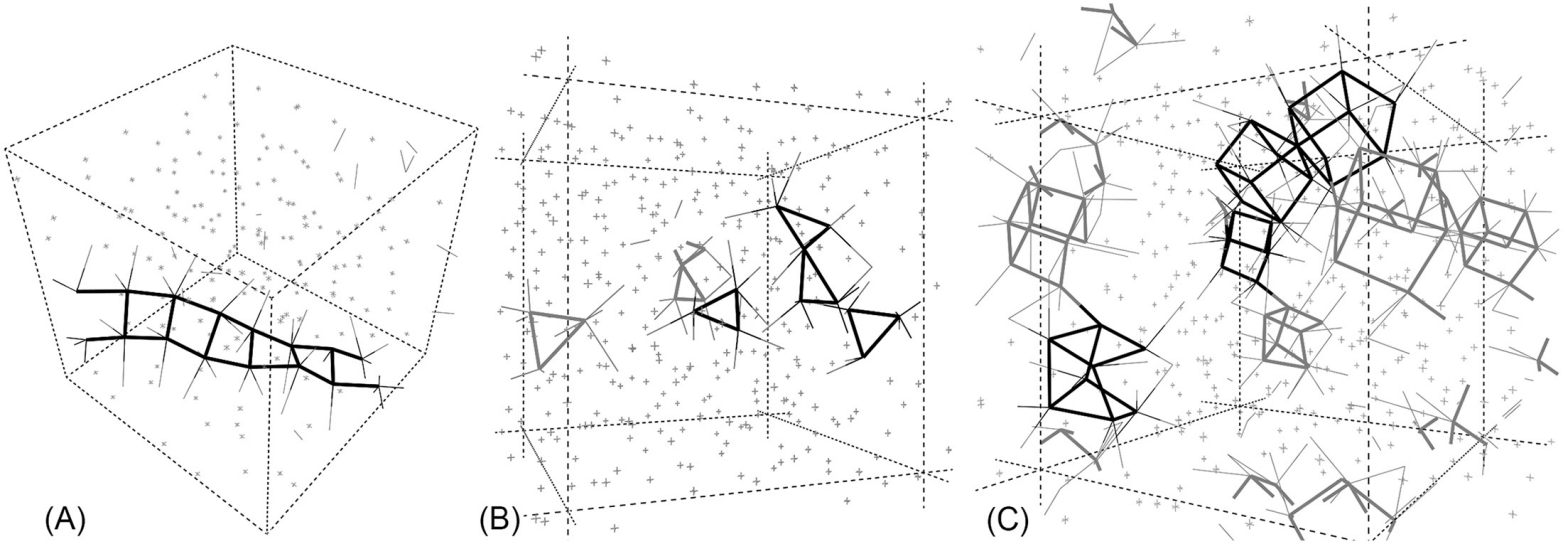
Some structures within *a-Bi*216. (A) Semi-planar structure associated with angles in the neighbourhood of 155°. (B) Triangular structures associated with the bump at 56.5°, reminiscent of quasi-equilateral triangles. (C) Deformed cubic-like arrangements reminiscent of the crystalline structure of bismuth, corresponding to 82°. Linked atoms are nearest neighbors.

As was done for the *g*(*r*), the PAD was obtained from the nuclei positions in the amorphous supercell with a program developed by U. Santiago [25]. The criterion assumed is that the atoms are bonded when they are located at distances ≤ 3.8 Å, which is the distance of the first minimum in the *a-Bi*216 PDF. This time the smoothing was done with a 3-point Fast Fourier filter using Origin 9. The peak positions in Fig 2 can be associated with some kind of geometrical arrangements: for example, some semi-planar structures can be associated with peaks in the neighbourhood of 155°, triangles can be associated with peaks near 56.5°, and cubic-like and square-like structures with peaks located near 82°, as shown in Fig 3.

As far as we know no other theoretical or computational result has been reported for amorphous bismuth and in what follows we present the vibrational and electronic densities of state obtained for the *a-Bi*216 structure that led to a PDF in agreement with experiment. Our accumulated simulational results indicate that the *undermelt-quench* method is a good approach for the generation of amorphous topologies of a variety of materials, semiconducting or metallic, elemental or alloyed [23, 26, 27]. Without sufficient experimental results for comparison we deposit our confidence in the realistic structures that we have generated using this approach.

### Electronic and vibrational properties

In Ref [28] the method of Ref [18] is applied to some model potentials, those having s-wave phase shifts only. Clearly the atomic topology, represented in the PDF, is relevant and so is the ensemble average over all possible topologies. These authors separate the components of the PDF into short and long range correlations and calculate both contributions to the eDOS. We do not use any of these approximations and the disorder that we take into account is the one represented by the PDF generated by our *undermelt-quench* procedure. To the best of our knowledge no electronic density of states (eDOS) for *a-Bi* has been measured or calculated, except for an attempt by Ley *et al*. [22]. They prepared an amorphous sample by argon-bombardment on a surface of mono-crystalline bismuth and claimed that the eDOS for crystalline and amorphous samples are almost the same. One has to wonder how much amorphous material is obtained by this ion bombardment method since for other cases, like amorphous silicon carbide prepared by similar methods, the experimental results are not consistent with the amorphous simulations [29]. Since films of *a-Bi* are usually obtained by depositing the liquid on very cold substrates and due to the mobility of these atoms, we cannot rule out the existence of some long range order in Ley’s amorphous sample which is prepared at room temperature; that may be why they conclude that the eDOS for crystalline and amorphous samples are very similar in shape. However for the crystalline phase there are experimental eDOS results and this will be another point of comparison to validate our calculations.

**Fig 4:**
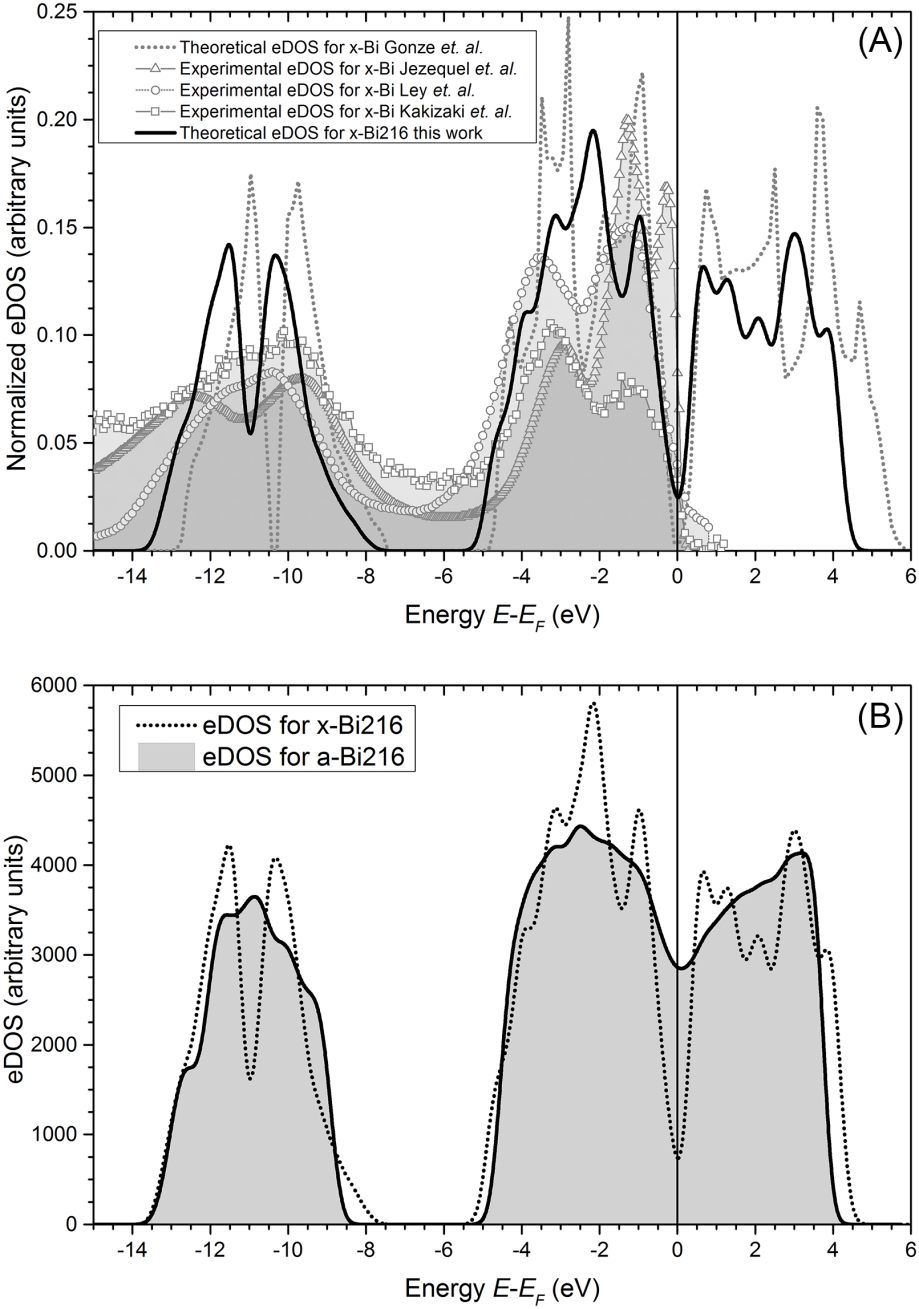
Electronic crystalline and amorphous behavior. (A) Comparison of our normalized result for *x-Bi*216 with the theoretical eDOS for *x-Bi* from Gonze *et al*. [32] and the experimental data taken from Jezequel *et al*. [30], Ley *et al*. [22] and Kakizaki *et al*. [31]. Normalization indicates that the area under each curve, up to the Fermi level, *E*_*F*_ − *E* = 0, is set equal to 1. (B) The calculated eDOS for the amorphous structure *a-Bi*216 (full line) and for the crystalline *x-Bi*216 (dotted line) are shown; the relevant feature for the amorphous is that the pseudo-gap has essentially disappeared and that therefore there are more electrons at *N* (*E*_*F*_) in *a-Bi* than in *x-Bi*.

In Fig 4A we show the eDOS calculated for *x-Bi*216 and compare it with the experimental data obtained by Angle-Resolved Photo Emission Spectroscopy (ARPES), X-ray Photoelectron Spectroscopy (XPS) and Ultraviolet Photoelectron Spectroscopy (UPS) from Jezequel *et al*. [30], Ley *et al*. [22] and Kakizaki *et al*. [31], respectively, as well as with the theoretical work of Gonze *et al*. [32]. The experimental techniques do not give the formal eDOS, but are quite useful tools for obtaining information about the electronic structures. In XPS, the energy distribution curves reflect the eDOS shape and in principle UPS spectra represent the joint eDOS and give precise information about the electronic structures of the valence band obtained from the excitation energy dependence of the energy distribution curves; so we can use those curves to validate our calculations. Two bands are present in the *x-Bi* eDOS, the s band (from -14 eV to -8 eV) and the p band (from -5 eV to 6 eV). For the s-band a split appears in all the theoretical and experimental results; also, for the p-band we get a split before the Fermi energy and this separation is a consequence of the spin-orbit coupling of the p orbitals [33]. Because *x-Bi* is a semimetal we get a pseudo-gap at the Fermi energy (Fermi level) in the theoretical results. Thus our calculated eDOS for the crystalline structure reproduces reasonably well the experimental eDOS and validates our calculations for amorphous bismuth.

Performing the same type of single-point energy calculation for the *a-Bi*216 structure we obtain the eDOS shown in Fig 4B. As for the *x-Bi* two bands can be identified, the s band and the p band but both smoother, i.e., the narrow peaks that appear in the crystalline results are now washed out due to a more homogeneous environment in the amorphous material. An interesting and relevant feature, is that the split peaks for the s- and the p-band have disappeared as has the pseudo-gap. In Fig 4B a substantial increment in the density of electronic states at the Fermi level, *N* (*E*_*F*_), for the amorphous is observed when the crystalline and amorphous results are compared, *i.e.*, for the amorphous supercell a fourfold increment (388%) in the density of states at the Fermi level appears with respect to the crystalline. The implications of such an outcome have to be handled with caution since in amorphous materials disorder causes localization of the electrons and although there are more states at the Fermi level, they may not be mobile. Nevertheless it is clear that there are more electrons at *N* (*E*_*F*_) in the amorphous than in crystalline bismuth. Could this imply that *a-Bi* is more metallic than *x-Bi*? Recent experimental results [34] corroborate this surmise, and indicate that despite the disorder in the amorphous there are non-localized electrons at the Fermi level.

**Fig 5:**
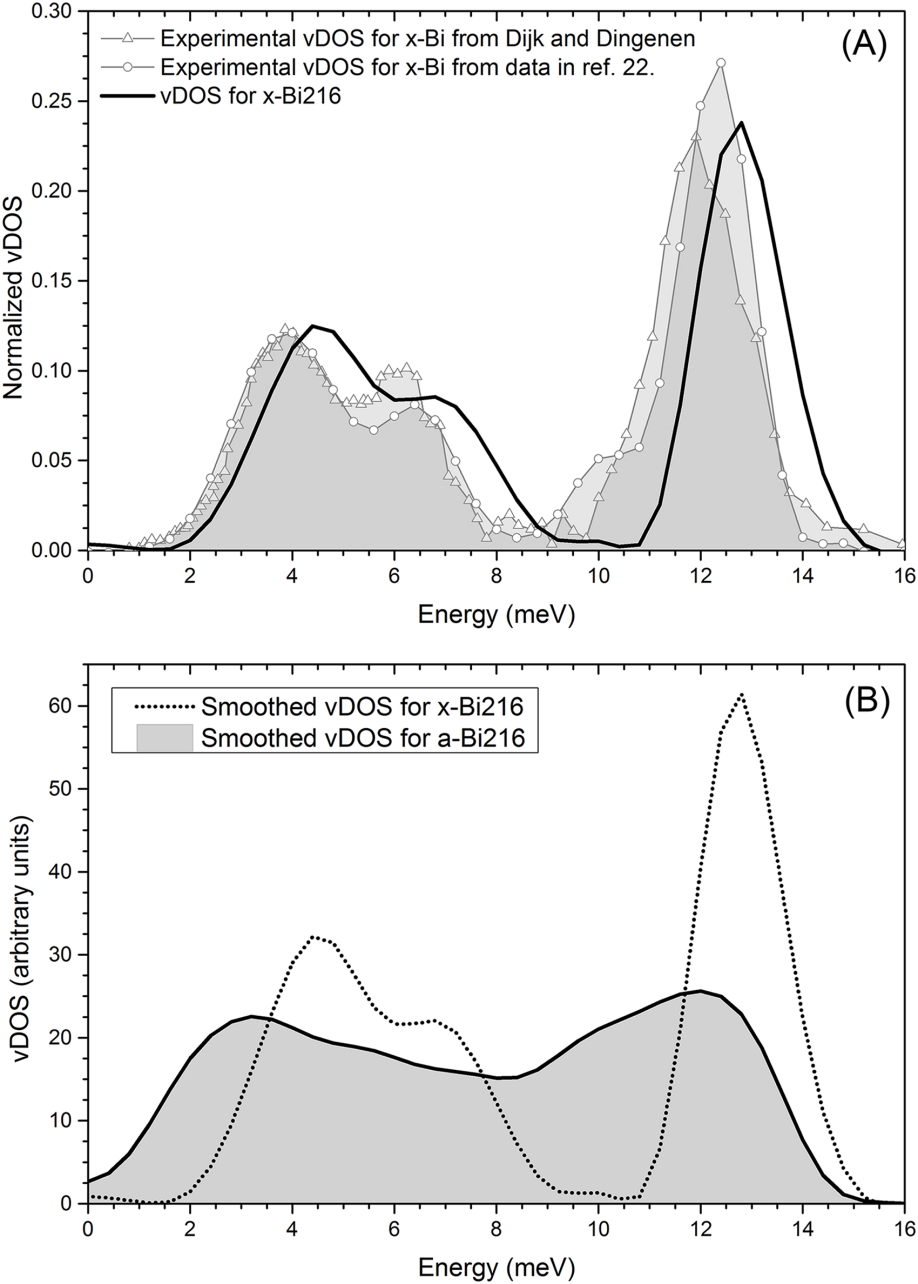
Vibrational behavior for the crystalline and the amorphous. (A) Comparison of the experimental vDOS (extracted and processed from Kakizaki [31] and taken from the results of Dijk and Dingenen [20]) and our computational calculations for *x-Bi*. The curves have been normalized so that the area under each curve is 1. (B) Comparison of the calculated vDOS for crystalline and amorphous supercells *x-Bi*216 and *a-Bi*216. The area under each curve has been set equal to 3*N*_*A*_.

Some authors, like Chen *et al*. [35] have experimentally determined the electron-phonon coupling function of *a-Bi* by McMillan’s inversion procedure [36, 37], but the pure vibrational density of states (vDOS or *F* (*ω*)) for the amorphous has never been reported. The experimental and calculated crystalline *F* (*ω*) shows two well-defined branches, the acoustic and the optical one. From the experimental results the acoustic branch is located between 1 and 8 meV, and the optical one between 8 and 14 meV (Fig 5A). The calculated results locate these branches between 2 and 9 meV and 10 and 15 meV, respectively. Our results are somewhat displaced towards higher energies, although the shapes of both curves are quite similar. We believe that our method reproduces experiment reasonably well and therefore that the result obtained for our amorphous sample is realistic. So far no experimental data for the *F* (*ω*) of *a-Bi* have been found. The *F* (*ω*) for the amorphous sample presents marked differences with respect to *x-Bi* (Fig 5B). First, we obtain the well-known presence between 0 and 4 meV of a large number of acoustic (soft) phonon modes generally reported for amorphous materials; next, the gap between the acoustic and optical branches, typical of crystalline samples with layer-like structures, disappears and a plateau appears. In fact, the whole distribution is shifted towards lower frequencies. Finally the extension of the vDOS on the energy scale is somewhat reduced when compared with the crystalline curve; see Fig 5B where a direct comparison between the two phases is presented.

We believe that these changes in the eDOS and the vDOS are related to the observed superconductivity in amorphous bismuth and its abscence in the crystalline phase. In what follows we will assess the adequacy of our results by using them to understand the relevant factors for superconductivity in bismuth

### Superconductivity in Bismuth

An approach to the superconducting properties of bismuth can be as complex as desired. One can adopt a simple BCS [38] approach or one can use the McMillan [36] approach which, being more complete, involves more parameters *ad-hoc* that may give a more detailed picture of the phenomenon. A simple BCS estimate of a possible crystalline superconducting transition temperature, if it exists, is presented first and a discussion of the implications in terms of McMillan’s approach follows.

#### A la BCS

The attractive interaction between electrons via phonons (e-ph) is fundamentally important for the occurrence of superconductivity and is related to the factor V in the BCS equation for the critical superconducting transition temperature *T*_*c*_. In the original form of the BCS theory *T*_*c*_ can be evaluated by

$$\begin{array}{c} T_{c}=1.13\theta_{D}\exp\left[-\frac{1}{N(E_{F})V}\right], \end{array}$$ (1)

where *N* (*E*_*F*_) is the electronic density of states at the Fermi level (*E*_*F*_) and *V* is the e-ph coupling potential. In Eq 1 we take *θ*_*D*_ = ℏ*ω*_*D*_/*k*_*B*_, with *ω*_*D*_ given by the following equation [39]:

$$\begin{array}{c} \omega_{D}=\exp\left[1/3+\frac{\int_{0}^{\omega_{\max}}\ln\left(\omega \right)F\left(\omega \right)d\omega }{\int_{0}^{\omega_{\max}}F\left(\omega \right)d\omega }\right]. \end{array}$$ (2)

This equation is assumed valid for *T* ≥ *θ*_*D*_ as stated by Grimvall [40]. The value of *ω*_*D*_ given by Eq 2 is an upper limit to the real value of the Debye frequency and this should be kept in mind when comparing with experiment. When *ω*_*D*_ is calculated using Eq 2 and our simulational results for *F*(*ω*) we find that the values for *θ*_*D*_ are 100 K for the amorphous and 129 K for the crystalline. The experimental values for *θ*_*D*_ reported by DeSorbo [41] for crystalline bismuth varies from 140 K at high temperatures to 120 K at low temperatures. Our calculations indicate that *θ*_*D*_ for the crystal lies between the experimental values. Grimvall [40] also mentions that *θ*_*D*_ for the amorphous is expected to be about 10% lower that the crystalline. Our results show that the amorphous is about 20% lower than the crystalline; so we can trust our calculations for *a-Bi*.

The presence of low frequency modes (low energy vibrations) in the *F*(*ω*) of the amorphous spectrum, and also the higher values of *α*^2^(*ω*) for low frequencies contribute to detonate the attractive phonon-mediated electron-electron interaction which is enhanced by the presence of additional electrons at the Fermi level in the amorphous phase.

Using Eq 1 and assuming that crystalline bismuth is a superconductor with transition temperature $T_{c}^{c}$, we can incorporate the changes in the eDOS at *N* (*E*_*F*_), in going from crystallinity to amorphicity, to obtain possible values for $T_{c}^{c}$ in terms of the amorphous transition temperature $T_{c}^{a}$ (More detailed description in the Specific Annotations section). If we now incorporate the two values of *θ*_*D*_ and assume that all other parameters are the same in both phases, we find that the value for the transition temperature for *x-Bi* is $T_{c}^{c}$ = 1.3 mK. This result is smaller than the 50 mK reported in Ref. [42]. This means that if superconductivity in the amorphous phase is caused mainly by the BCS parameters eDOS and vDOS and if the coupling e-ph is assumed to be the same for both phases, then the crystalline phase may become superconducting with a transition temperature of 1.3 mK.

Assuming that the e-ph interaction is the same for the amorphous and crystalline phases is a strong assumption, as it is assuming that both phases are weak coupling and can be described by the BCS formalism. But, how relevant are these assumptions? Quite so. In the first place, it is experimentally known that the amorphous phase is strong coupling [43] whereas the crystalline one has not even been found yet to superconduct. Secondly, if *x-Bi* has not been found to be a superconductor, then the attractive e-e potential for this phase may be smaller than for the *a-Bi*. Since the surface *T*_*c*_ *vs* (*N*(*E*), *V*) is monotonic, when (*N*(*E*), *V*) decreases, so does the corresponding *T*_*c*_; therefore if the e-ph interaction is weaker in *x-Bi* one should expect a lower crystalline transition temperature, *i. e.*, *T*_*actual*_<<1.3 mK. Thus our estimation should be considered to be an upper bound for this phase. Next we investigate this point using the McMillan formalism.

#### A la McMillan

Assuming that *x-Bi* may be described by McMillan’s formula [36, 37] we calculate the corresponding parameters to obtain an estimation of the electron-phonon interaction, or equivalently, of the strength of the parameter *λ*. An experimental result for *λ* for *a-Bi* using tunnelling experiments leads to *λ*^*a*^ = 2.46 [35]. Contrary to the assumption used in the previous section about the constancy of *V* for both phases, we would expect the electron-phonon coupling to be more preponderant in the amorphous phase than in the crystalline one; however we cannot calculate it for the crystal and therefore we shall estimate it. First we consider that the coupling function *α*^2^(*ω*) is the same for both phases. In order to see the relevance of *λ* in this formalism we do the following. The parameter *λ* can be calculated in terms of *α*^2^(*ω*) and *F*^*c*^(*ω*) as follows,

$$\begin{array}{c} \lambda^{c}=2\int_{0}^{\infty }\omega^{-1}\alpha^{2}\left(\omega \right)F^{c}\left(\omega \right)d\omega \;; \end{array}$$ (3)

the result is *λ*^*c*^ = 0.98. This implies that if, in fact, the value of *λ*^*c*^ is less than 1, then *x-Bi* should be in the weak coupling regime if it is a superconductor. This also corroborates the assumption of the preponderance of the e-e attraction in the amorphous with respect to the crystalline.

The relationship among *T*_*c*_, *θ*_*D*_, μ* and *λ* in the McMillan formalism is given by the equation

$$\begin{array}{c} T_{c}=\frac{\theta_{D}}{1.45}\exp\left[\frac{-1.04(1+\lambda )}{\left(\lambda -\mu^{*}\left(1+0.62\lambda \right)\right)}\right] \end{array}$$ (4)

where μ* is the Coulomb pseudopotential.

**Fig 6:**
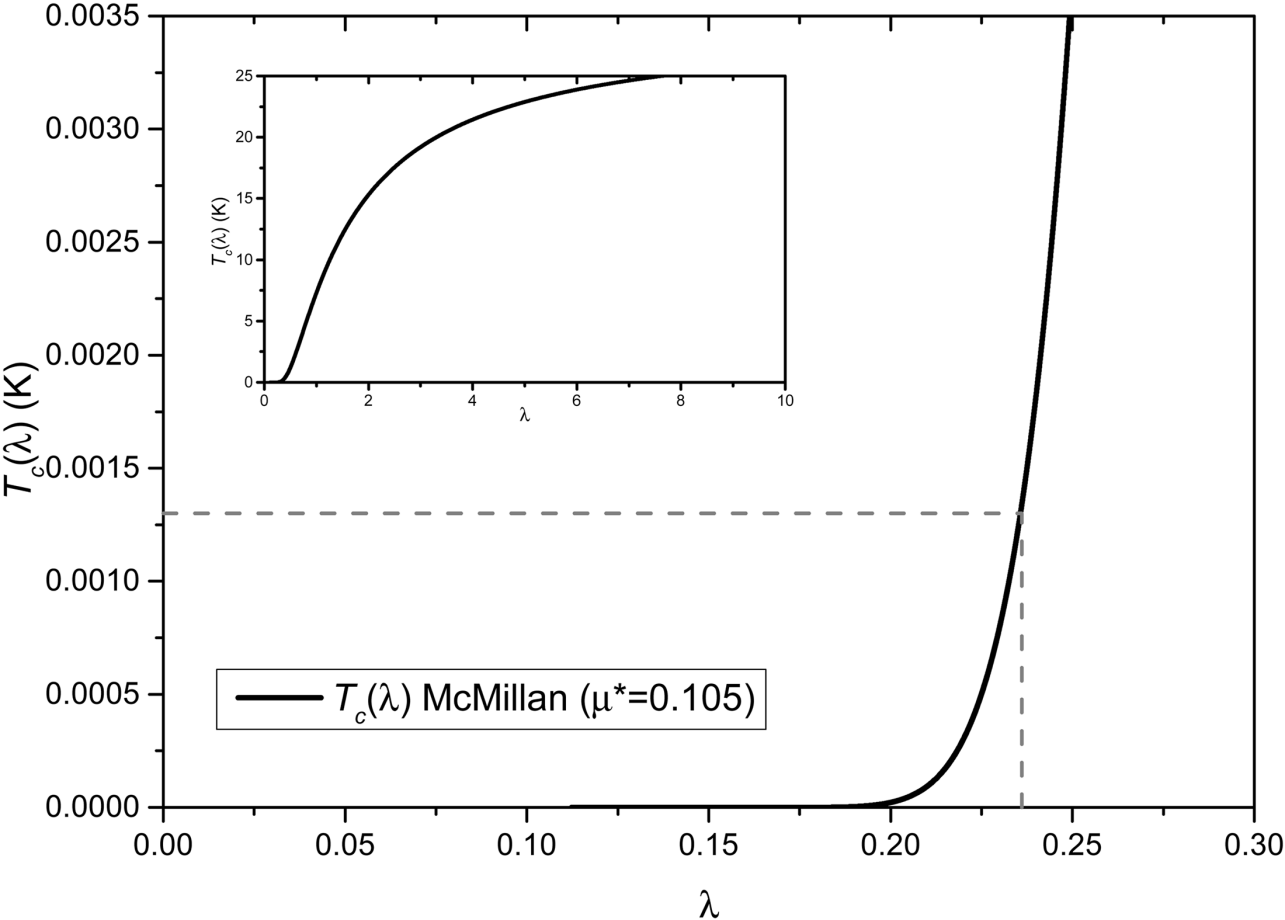
Dependence of *T*_*c*_(*λ*) on *λ* from McMillan’s Eq 4 for *θ*_*D*_ = 129K. The behavior indicates that the *T*_*c*_ dependence on *λ* is not very strong for high values of *λ*. For low values, *T*_*c*_ changes rapidly with *λ*. The inset is an extended plot. The dotted lines show the value of *λ* (0.236) for the estimated *T*_*c*_.

A plot of *T*_*c*_(*λ*) *vs*. *λ* appears in Fig 6, where a value of μ* = 0.105 is used. The behavior indicates that the *T*_*c*_ dependence on *λ* is not very strong for high values of *λ*. For low values, *T*_*c*_ changes rapidly with *λ* and we shall focus on this region. For the superconducting transition temperature estimated in the BCS formalism, 1.3 mK, the *λ* obtained from Fig 6 is 0.236. This clearly indicates that the e-ph coupling is much stronger in the *a-Bi* than in *x-Bi*, 2.46 *vs*. 0.236, which implies that the coupling constant for the amorphous is more than 11 times as intense as for the crystalline. This in turn implies that our assumption of the similarity of the strength of the electron-phonon interaction in both *x-Bi* and *a-Bi* used earlier should be handled with care and the estimated transition temperature of 1.3 mK should be considered, at best, an upper limit for the crystalline material as mentioned above.

## Specific Annotations

### The Structure Simulations

#### The modified *undermelt-quench* process

To generate the structure of amorphous bismuth *a-Bi* we start with a 216-atom diamond-like supercell with an edge length of 19.7 Å, a volume of 7645.37 Å^3^ and periodic boundary conditions, *a-Bi216*. Since *x-Bi* has a rhombohedral structure, the diamond-like structure is unstable and will disorder readily as the temperature is raised. At temperatures just below the melting point the structure becomes non-crystalline but upon cooling has the tendency to crystallize so the time step has to be chosen adequately to avoid this change of phase. Since this supercell of Bi is unstable, during the Molecular Dynamics (MD) process the structure disarrays easily and then finds a metastable amorphous point. We choose interatomic distances on the initial structure that are close to the real bond lengths and a density close to the solid crystalline bismuth (9.8 g/cm^3^). The computational procedure is performed with the code FASTSTRUCTURE that uses the Harris functional based Local Density Approximation (LDA) with the Lin-Harris approach to perform Density Functional Theory (DFT) calculations of the system’s energy and then get the forces that act on the atoms during the MD process. To solve the interactions in the system a linear combination of atomic orbitals (LCAO) is used, so a set of basis functions are needed; the Vosko Wilk and Nusair (VWN) approximation is incorporated. To guarantee that the interatomic interactions are adequately represented the cutoff of the orbital functions is set to 5 Å, enough to include second neighbours in the amorphous structure (fourth neighbours in the crystalline structure).

**Fig 7:**
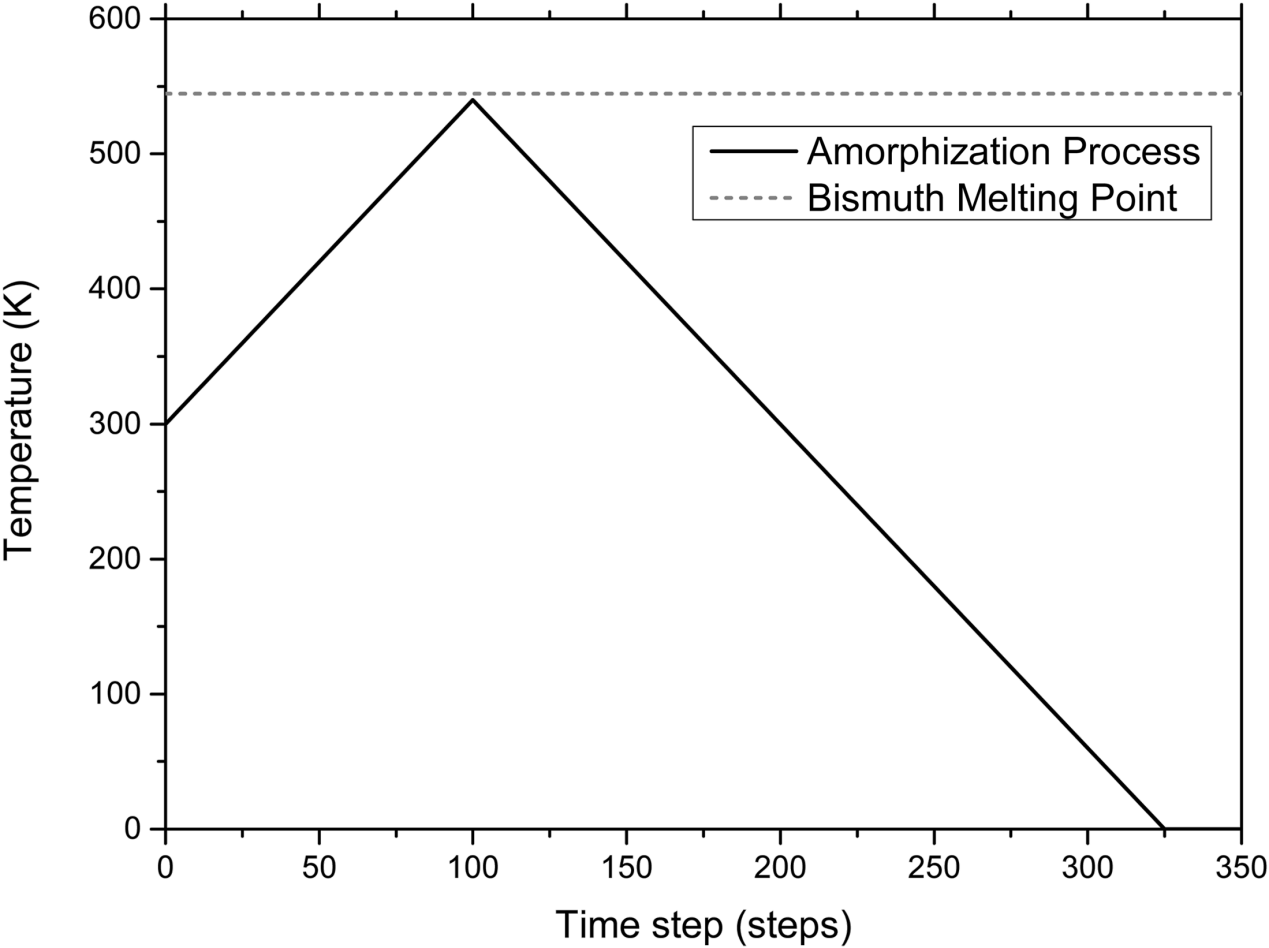
The modified *undermelt-quench* process. This is the amorphization process used to generate amorphous bismuth supercells. The heating ramp stops just below the melting point whereas the cooling ramp has the same (negative) slope.

**Fig 8:**
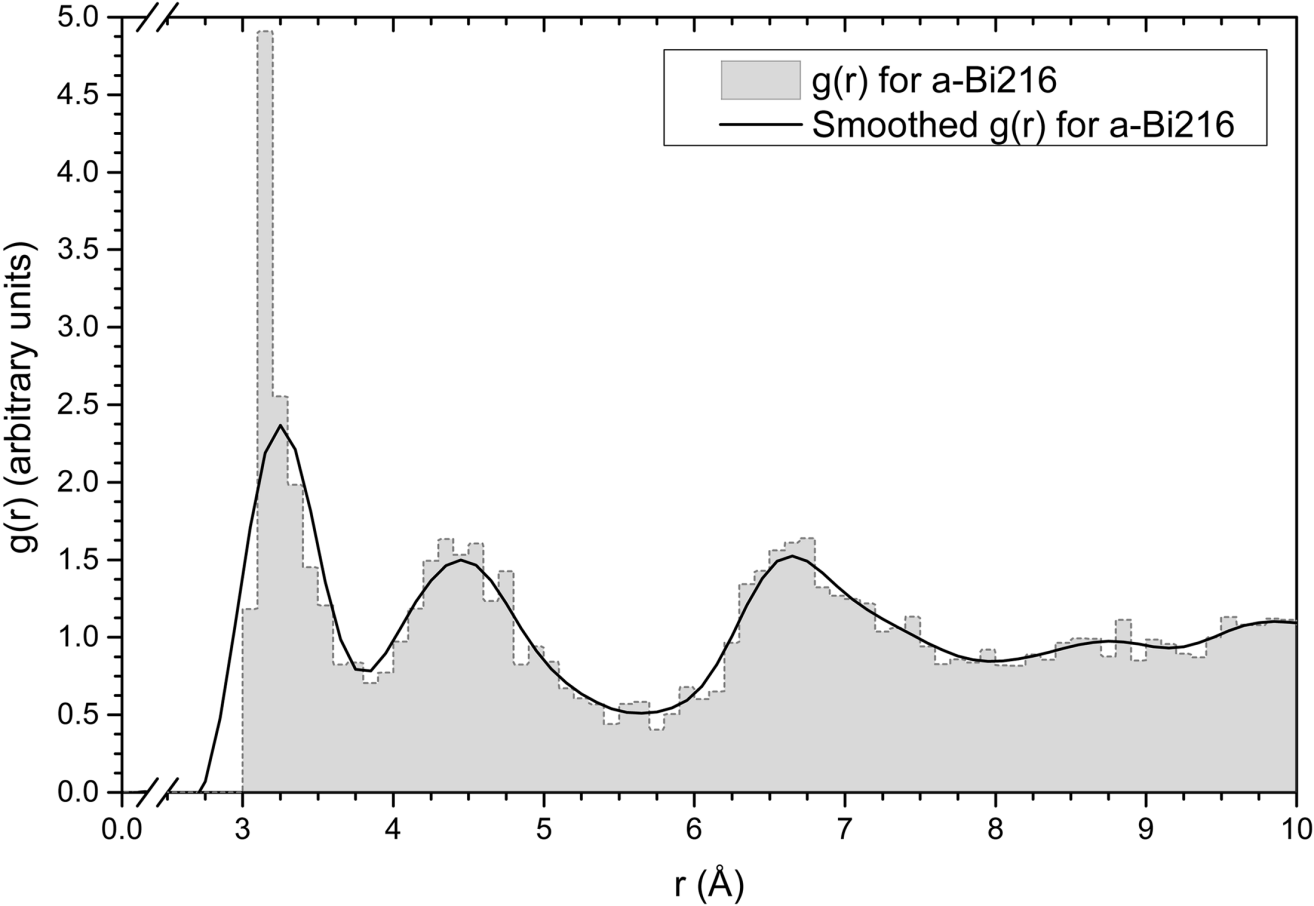
The amorphous PDF (or *g*(*r*)). The figure shows the amorphous *g*(*r*) (grey filled figure) and its 2-point smoothed curve (continuous black line) for *a-Bi216*.

The computational process to amorphize structures is called the *undermelt-quench* approach and it has been shown that the final structures agree well with experiments for other simulations. Originally the process consisted first of heating the supercell to just below the melt (undermelt), then cooling the system down to close to 0 K (quench), and finally using a set of thermal panels to relax the stresses that appear in the sample. Since originally this method was applied successfully to semiconductor materials a slight modification had to be incorporated when generating *a-Bi*, *i. e.*, only the undermelt and the quench panels are present. The initial supercell is heated from 300 K to 540 K (4.5 K below the bismuth melting point) in 100 computational steps. Then it is quenched from 540 K to close to 0 K in 225 steps to maintain the same absolute thermal rate for the heating and the cooling processes, see Fig 7. The time step that inhibits the amorphous from becoming crystalline is 26.88 fs, so the time lapse of the total simulated thermal procedure is about 10 ps. Once the processes are finished, a geometry optimization is performed to guarantee that the energy of the structure is a minimum, representing a metastable amorphous solid. In all cases periodic boundary conditions are incorporated. Once the structure is obtained the Pair Distribution Function (PDF or *g*(*r*)) can be calculated from the atomic positions in the model but since the number of atoms is small the PDF needs to be smoothed to emulate a large number of atoms, a common procedure. This is done with a 2-point Fast Fourier smoothing in Origin 9 shown in Fig 8. The smoothing weight was chosen so that the peaks of our calculations have the same height as those of the experimental PDF of Fujime [2].

### Electronic and vibrational simulations

**Fig 9:**
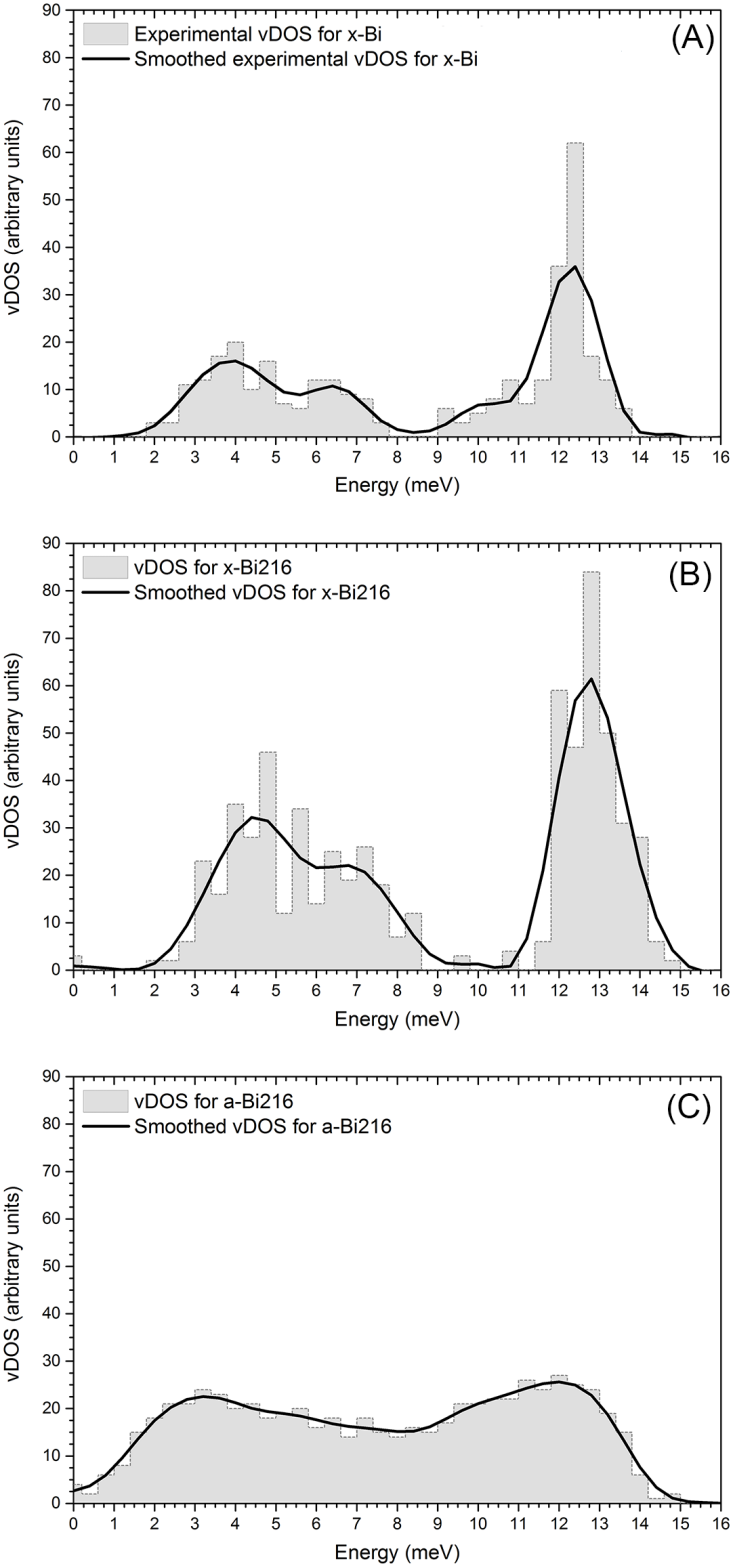
The vibrational distributions vDOS (or *F*(*ω*)). The figures show the vDOS (grey filled figures) and their 3-point smooth curves (continuous black lines) for (A) the experimental *x-Bi* (10) (B) the theoretical *x-Bi216* calculated in this work and (C) the theoretical *a-Bi216* calculated in this work.

**Fig 10:**
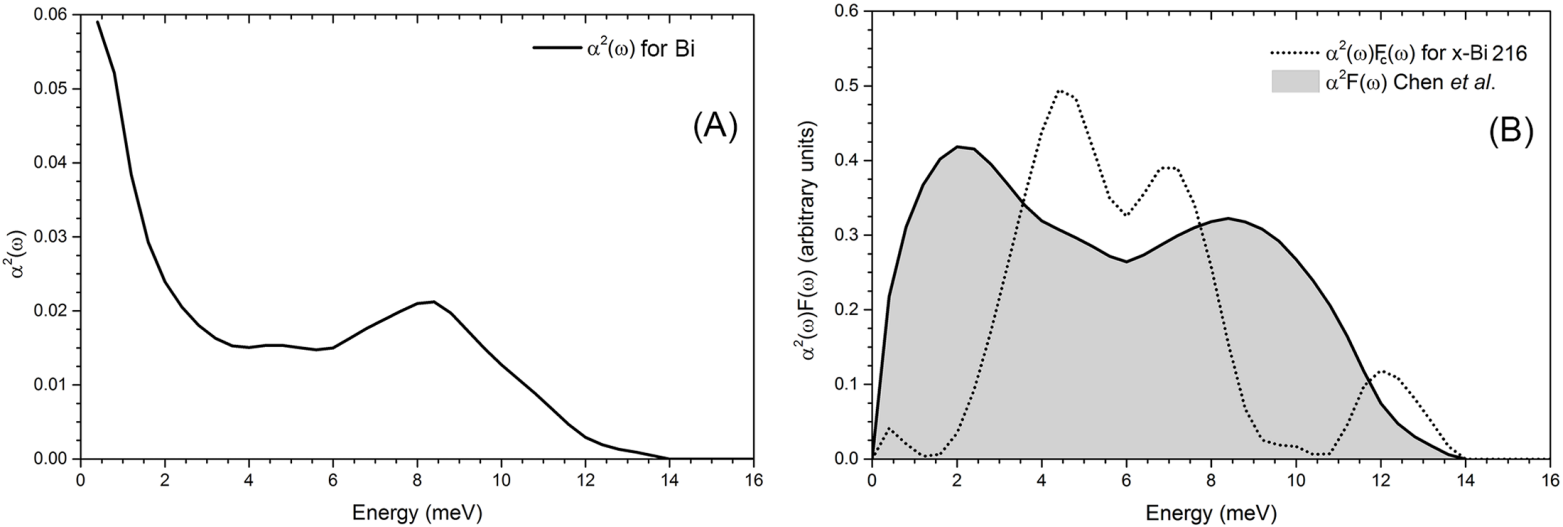
The electron-phonon coupling function for Bi. (A) *α*^2^(*ω*) for *a-Bi* obtained from the ratio between the experimental data of Chen *et al*. [35] and our calculated *F*_*a*_(*ω*) for amorphous bismuth. That is, the result of dividing Chen’s experimental data by our simulational calculations for *F*_*a*_(*ω*) point by point. (B) *α*^2^(*ω*)*F*(*ω*) calculated from the *α*^2^(*ω*) obtained in (A) and the crystalline *F*_*c*_(*ω*) from this work, compared with Chen’s experimental results for *a-Bi*.

In order to obtain the eDOS from the final amorphous structure, we perform a single-point energy calculation using the DMol^3^ code [15, 16] with finer parameters than for the structural calculations. A double-numeric basis set is used and also a fine mesh, with the VWN [11] correlation energy incorporated. The unrestricted spin polarization approach is used to describe the electronic structure. For these calculations the Density-functional Semi-core Pseudo Potential (DSPP) approximation is used so that most of the electronic structure of the bismuth atoms is incorporated through this pseudopotential, without burdening the computational calculations. The electronic calculations were performed at the supercell Gamma point since no symmetries were involved. To compare the experimental vDOS for crystalline bismuth (*x-Bi*) from Ref. [19] with the crystalline 216-atom bismuth supercell (*x-Bi216*, see Fig 9B) we digitize the experimental phonon dispersion curves and apply a frequency count with a bin width of 0.4 meV. Then applying a 3-point Fast Fourier smoothing in Origin 9 on this data and normalizing the smoothed curve to 3 times the number of atoms we obtain the corresponding experimental vDOS (see Fig 9A). The same procedure is applied to the data obtained from the frequency calculations done on the *a-Bi216* supercell (see Fig 9C) and the same parameters are used allowing us to handle the curves to obtain *α*^2^(*ω*) for amorphous bismuth. To obtain *α*^2^(*ω*) we digitize the experimental *α*^2^(*ω*)*F*(*ω*) from Chen *et al*.[35] at intervals of 0.4 meV. Then we take the ratio, point by point, of *α*^2^(*ω*)*F*(*ω*)/*F*_*a*_(*ω*) where *F*_*a*_(*ω*) (vDOS) is the curve calculated in this work for *a-Bi216*. In this manner we obtain *α*^2^(*ω*) which is shown in Fig 10A. Once *α*^2^(*ω*) is obtained, and assuming that it is the same for the crystalline phase, we calculate *α*^2^(*ω*)*F*_*c*_(*ω*) for crystalline bismuth by multiplying point by point this *α*^2^(*ω*) by *F*_*c*_(*ω*), the crystalline vDOS obtained in this work.

### A simple superconductivity analysis

For the following analysis we assume that bismuth is a BCS superconductor and we incorporate the changes in the eDOS at the Fermi level *N* (*E*_*F*_) in going from crystal to amorphous. The consequences of these changes in the BCS equation (Eq 1 in the main text) can now be estimated. Assuming that superconductivity is possible for *x-Bi*, with a superconducting transition temperature $T_{c}^{c}$, and that *V* is essentially the same for the amorphous and crystalline phases, we can then estimate $T_{c}^{c}$ in terms of $T_{c}^{a}$ as follows. The BCS equation for the crystalline material is

$$\begin{array}{c} T_{c}^{c}=1.13\theta_{D}^{c}\exp\left[-{1/N^{c}\left(E_{F}\right)V\;}^{c}\right], \end{array}$$ (5)

and for the amorphous phase the equation becomes

$$\begin{array}{c} T_{c}^{a}=1.13\theta_{D}^{a}\exp\left[-1/4N^{c}\left(E_{F}\right)V^{a}\;\right], \end{array}$$ (6a)

$$\begin{array}{c} {\left(T_{c}^{a}\right)}^{4}={\left(1.13\theta_{D}^{a}\right)}^{4}{\left\{\exp\left[-1/4N^{c}\left(E_{F}\right)V^{a}\right]\right\}}^{4}, \end{array}$$ (6b)

since 4*N*^*c*^(*E*_*F*_) ≈ *N*^*a*^(*E*_*F*_). Taking the ratio ${T_{c}^{c}/\left(T_{c}^{a}\right)\;}^{4}$, using Eqs 5 and 6b, the following is obtained,

$$\begin{array}{c} \frac{T_{c}^{c}}{{\left(T_{c}^{a}\right)}^{4}}=\frac{1.13\theta_{D}^{c}}{{\left(1.13\theta_{D}^{a}\right)}^{4}}, \end{array}$$ (7)

and if we accept that $1.3\theta_{D}\approx \theta_{D}^{c}$ as calculated in this work (see main text), the expression becomes

$$\begin{array}{c} T_{c}^{c}=\frac{1.3{\left(T_{c}^{a}\right)}^{4}}{{\left(1.13\theta_{D}^{a}\right)}^{3}}. \end{array}$$ (8)

Since $T_{c}^{a}$ is 6.2 K, the experimental result, Eq 8 gives a crystalline transition temperature of $T_{c}^{c}=1.32\times 10^{-3}\text{K}$.

## Conclusions

We report the computer generation of amorphous bismuth that displays pair distribution functions for a 216-atom supercell in agreement with experiment. Assuming this agreement is an indication of the representativeness of our amorphous structure, we then proceed to calculate the electronic and vibrational densities of states for both amorphous and crystalline supercells. These results suggest that the marked differences found are responsible for the radically different superconducting properties. Estimations of a transition temperature for the crystalline, assuming that the relevant factors are the eDOS and vDOS leads us to predict an upper bound for the superconducting transition temperature of the order of 1.3 mK. If the strength of the electron-phonon coupling, as determined using McMillan’s approach, is incorporated then the upper limit will be depleted and one expects *T*_*c*_(*λ*) ≪ 1.3 mK. This may explain why the crystalline superconductivity has not been observed yet.

Features to be noted are that the electronic density of states of the amorphous is about 4 times larger than the crystalline at the Fermi level, and that the vibrational density of states of the amorphous develops a noticeable accumulation of phonon modes at low frequencies along with the disappearance of the “gap” at intermediate frequencies. One may also argue that the increment of *N* (*E*_*F*_) in the amorphous may not be relevant for the mobility of the electrons which could be inhibited by the disorder that fosters a localization phenomenon in this phase, or that not all low frequency modes in the amorphous will contribute to the coupling *α*^2^(*ω*) since there may also be some localization of the vibrational states. However recent experimental results [34] indicate that the transition from crystallinity to amorphicity entails a transition from semimetallic to metallic behaviour which points to the fact that the disorder in the amorphous does not hinder this transition.

To seal the discussion, dealing with amorphous materials is not an easy task. Concepts like localization, soft-phonon modes and ill-defined electronic and vibrational states have to be handled with care because *q* and *k* are not good quantum descriptors. Since BCS does not rely explicitly on periodic (crystalline) assumptions, it can be applied to amorphous superconductors. When one attempts to apply McMillan’s equation to disordered materials there are conceptual and practical difficulties. Nevertheless these limitations do not seem to be applicable to this work.
